# The involvement of Nrf2 in the protective effects of (-)-Epigallocatechin-3-gallate (EGCG) on NaAsO_2_-induced hepatotoxicity

**DOI:** 10.18632/oncotarget.18582

**Published:** 2017-06-21

**Authors:** Xiao-Dong Han, Yan-Yan Zhang, Ke-Lei Wang, Yong-Pan Huang, Zhong-Bao Yang, Zhi Liu

**Affiliations:** ^1^ Department of Anesthesia, The Second Affiliated Hospital, Xi’an Jiaotong University, Xi’an, Shaanxi 710049, China; ^2^ Medical College, Yan’an University, Yan’an, Shaanxi 716000, China; ^3^ Department of Anesthesia, Xianyang Rainbow Hospital, Xianyang, Shaanxi 712021, China; ^4^ Department of Pharmacology, Guizhou Medical University, Guiyang, Guizhou 550004, China; ^5^ Department of Pharmacology, Guiyang Nursing Vocational College, Guiyang, Guizhou 550025, China; ^6^ Department of Pharmacology, School of Medicine, Hunan Normal University, Changsha, Hunan 410013, China; ^7^ Xiangya School of Medicine, Central South University, Changsha, Hunan 410078, China; ^8^ Department of Pharmacy, Affiliated Changsha Hospital of Hunan Normal University, Changsha, Hunan 410006, China; ^9^ Department of Urology Surgery, The Second Affiliated Hospital of Guizhou Medical University, Kaili, Guizhou 556000, China

**Keywords:** NaAsO_2_, (-)-Epigallocatechin-3-gallate (EGCG), hepatotoxicity, oxidative stress, nuclear factor erythroid 2-related factor 2 (Nrf2)

## Abstract

Arsenic exposure produces hepatotoxicity. The common mechanism determining its toxicity is the generation of oxidative stress. Oxidative stress induced by arsenic leads to the activation of nuclear factor erythroid 2-related factor 2 (Nrf2) pathway. (-)-Epigallocatechin-3-gallate (EGCG) possesses a potent antioxidant capacity and exhibits extensive pharmacological activities. This study aims to evaluate effects of EGCG on arsenic-induced hepatotoxicity and activation of Nrf2 pathway. Plasma activities of alanine aminotransferase, aspartate aminotransferase, alkaline phosphatase, and lactate dehydrogenase were measured; Histological analyses were conducted to observe morphological changes; Biochemical indexes such as oxidative stress (Catalase (CAT), malonyldialdehyde (MDA), superoxide dismutase (SOD), glutathione (GSH), reactive oxygen species (ROS)), Nrf2 signaling related genes (*Nrf2, Nqo1, and Ho-1*) were assessed. The results showed that EGCG inhibited arsenic-induced hepatic pathological damage, liver ROS level and MDA level. Arsenic decreases the antioxidant enzymes SOD, GPX, and CAT activity and the decrease was inhibited by treatment of EGCG. Furthermore, EGCG attenuated the retention of arsenic in liver tissues and improved the expressions of Nrf2 signaling related genes (*Nrf2, Nqo1, and Ho-1*). These findings provide evidences that EGCG may be useful for reducing hepatotoxicity associated with oxidative stress by the activation of Nrf2 signaling pathway. Our findings suggest a possible mechanism of antioxidant EGCG in preventing hepatotoxicity, which implicate that EGCG may be a potential treatment for arsenicosis therapy.

## INTRODUCTION

Inorganic arsenic is a major environmental pollutant with multiple toxic effects in animal and human populations [1, 2]. Arsenic at low levels represent the major source of arsenic exposure and tend to accumulate in the food chain worldwide [3]. Arsenic contamination may occur due to its industrial uses in the production of agricultural pesticides, glass production, and in medicine [4]. Arsenic entering to human body may lead to exceed the permissible limit and cause serious toxicity to human body. Arsenic causes marked damage in various organs, including the liver [5]. It has been demonstrated that arsenic leads to hepatocellular injury, fatty degeneration and progressive fibrosis [1]. However, the molecular mechanisms of hepatotoxicity caused by arsenic are not fully unclear. At present, emerging evidences have indicated that toxic manifestations of arsenic are mainly attributed to oxidative stress [6–8]. It has widely been accepted mechanism to explain the toxicity induced by arsenic [8]. Liver is regarded as target organ for metabolism and detoxification of arsenic. Consequently, in order to reduce arsenic-induced oxidative stress, the development of effective therapeutic strategies is urgently needed. Medications with antioxidants are potentially to decrease the hepatotoxicity induced by arsenic.

Nuclear factor erythroid 2-related factor 2 (Nrf2), a member of the cap’n’collar family of bZIP transcription factors. Nrf2 is activated in response to oxidative stress and toxic damages *in vivo* and *in vitro*. Under homeostatic conditions, Nrf2-dependent transcription is modulated by Kelch-like ECH-associated protein 1 (Keap 1) in the cytoplasm. In response to oxidative or electrophilic stress, keap 1 dissociates, then Nrf2 translocates into the nucleus and activates its downstream’s target genes, resulting in the up-regulation of detoxifying enzyme and antioxidantproteins such as Hmox-1, NQO-1, glutathione transferase (Gst), and glutathione (GSH) [9, 10]. Recent studies have indicated that activation of the Nrf2 pathway could alleviate numerous liver disorders.

(-)-Epigallocatechin-3-gallate (EGCG) is the most abundant and active polyphenol in green tea and widely consumed as an antioxidant. The biologic properties of EGCG has been reported that such as being antioxidant hepatoprotection and studies have indicated the protective role of EGCG in many diseases including cancer and cardiovascular diseases [11]. These mentioned diseases associated with the putative benefits of EGCG is linked to its strong free radical scavenging and antioxidant properties. Some studies have suggested the ameliorating effect of EGCG against metal-induced hepatotoxicity *in vitro* model [12]. However, the mechanism by which EGCG elicits hepatoprotective and antioxidant effects in association with Nrf2 is unclear. Therefore, the aim of the present study was to evaluate the protective effects of EGCG on arsenic-induced hepatic oxidative injury and to elucidate the mechanisms underlying these protective effects.

## RESULTS

### EGCG alleviated NaAsO_2_-induced liver damage *in vivo* and *in vitro*

**Figure 1:**
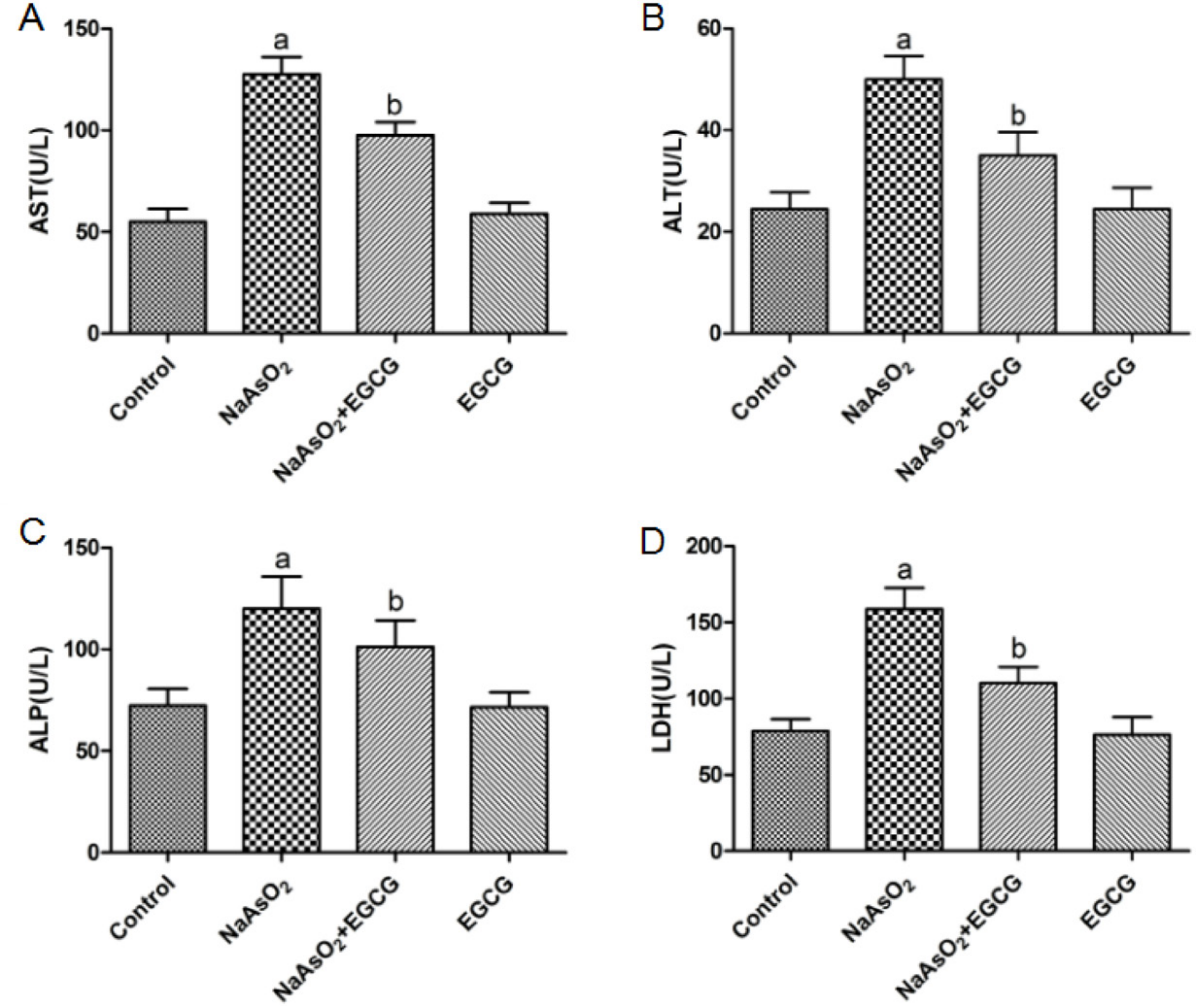
Effects of NaAsO_2_ and/or EGCG administration on indices related to hepatotoxicity in rats **(A)** AST, **(B)** ALT, **(C)** ALP, **(D)** LDH. Data are represented as the means ± SEM. Vs control, ^a^*P*<0.05;Vs NaAsO_2_ group, ^b^*P*<0.05.

It is reported that the activities of alanine aminotransferase (ALT), aspartate aminotransferase (AST), alkaline phosphatase (ALP), and lactate dehydrogenase (LDH) reflect the integrity of plasma membranes in liver tissue and hepatocytes. To investigate the effect of EGCG on NaAsO_2_-induced liver injuries, the concentration of these cytoplasmic enzymes were tested in liver tissue. As shown in Figure 1, the concentrations of ALT, AST, ALP and LDH in liver tissue increased markedly in rats, which treated with NaAsO_2_ throughout the experiment. Treatment with EGCG significantly decreased the levels of those enzymes induced by NaAsO_2_. Meanwhile, administration of EGCG alone did not affect those enzyme activities.

**Figure 2:**
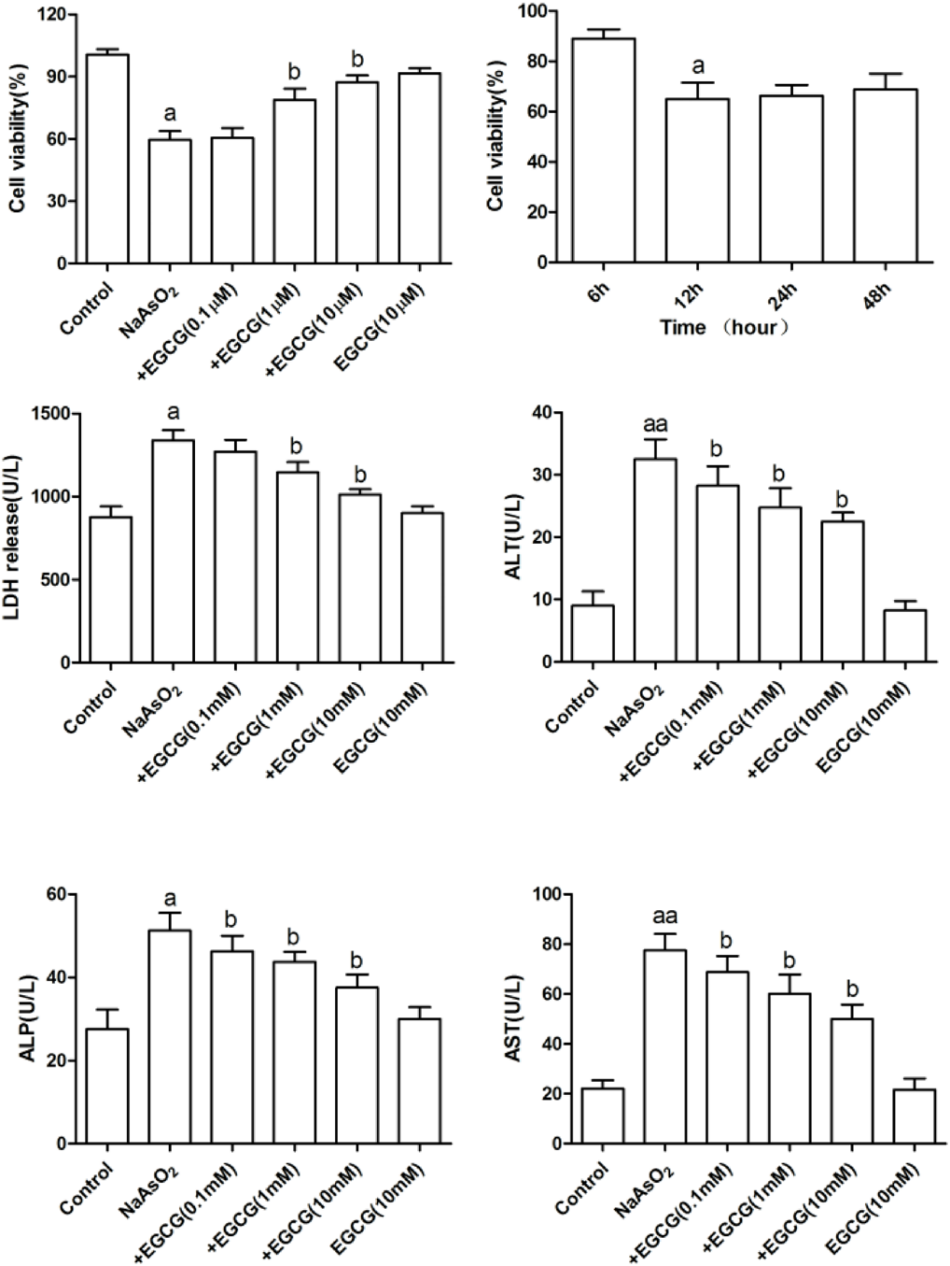
Effects of EGCG on NaAsO_2_-induced hepatocytes damage and oxidative stress Data are represented as the means ± SEM. Vs control, ^a^*P*<0.05, ^aa^*P*<0.01;Vs NaAsO_2_ group, ^b^*P*<0.05.

To further elucidate the protective mechanism of EGCG on NaAsO_2_-induced injury in hepatocytes, the levels of ALT, AST, ALP, and LDH were also investigated. As shown in Figure 2, an increase in ALT, AST, ALP, and LDH were detected exposure to NaAsO_2_. However, treatment with EGCG, these enzymes were dramatically decreased dose-dependently.

**Figure 3:**
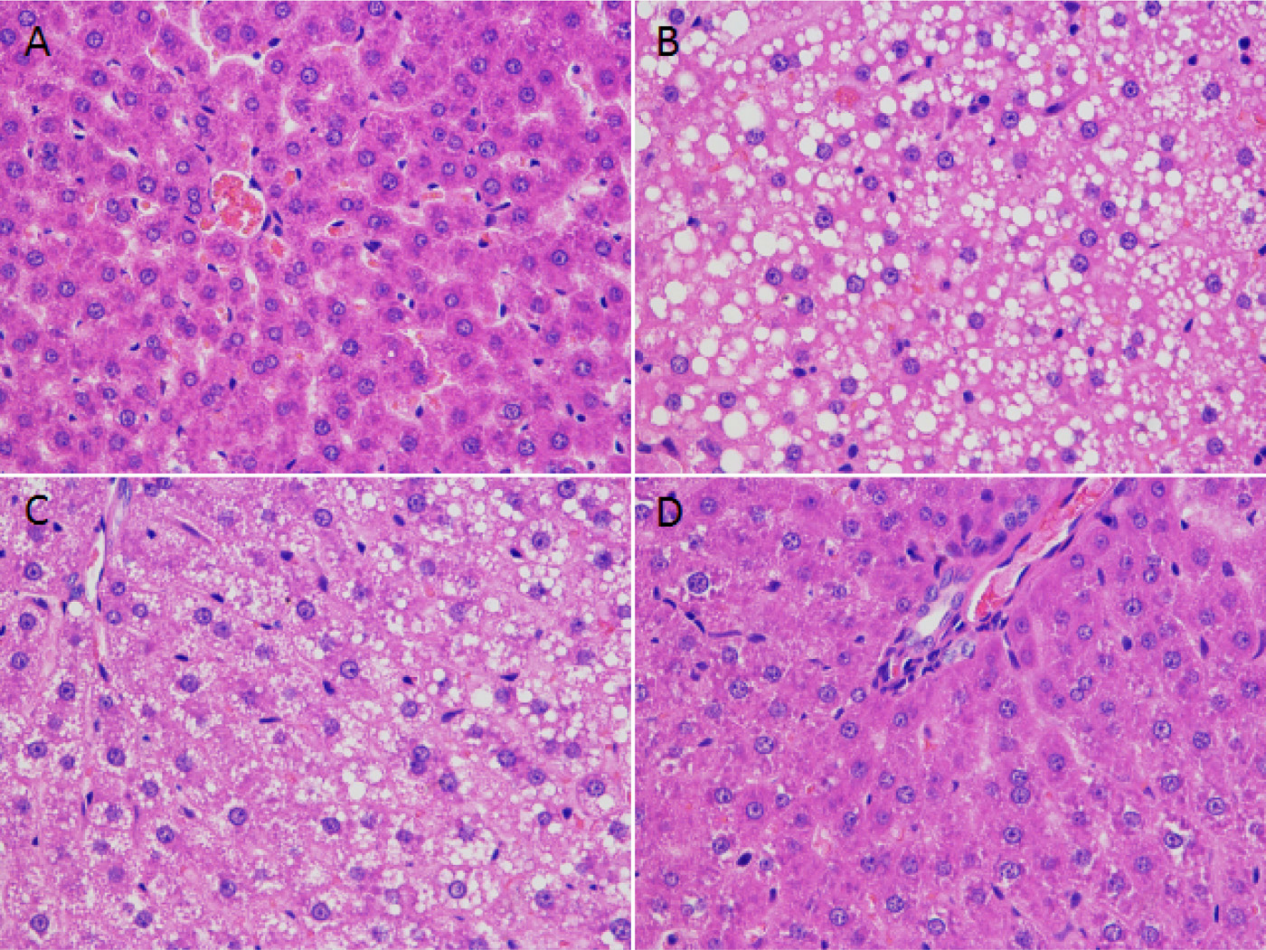
Morphological changes in mouse liver after NaAsO_2_ and/or EGCG **(A)** Control group, normal structure of hepatic cord, hepatic sinusoid, and hepatocyte. **(B)** NaAsO_2_ group, the basic performance of the hepatocyte poisoning could be found in NaAsO_2_ treatment group, such as dim boundary of hepatocyte, dismissed cell membrane, cytoplasm disintegrating pieces, and the accumulation of lipid droplets intracytoplasm; meanwhile, the specific hepatocyte balloon degeneration performance can be observed. At the same time, inflammatory cells infiltration scattered in the liver tissue. **(C)** NaAsO_2_+EGCG group, fatty degeneration of hepatocytes and congestion but not observed balloon degeneration and hepatocyte disintegration. **(D)** EGCG group, normal structure of liver cells as control group (200×).

To further confirm the protective effect of EGCG on NaAsO_2_-induced liver damage, we examined liver histology in tissues from the treated mice. Arsenic exposure resulted in dim boundary of hepatocyte, dismissed cell membrane, cytoplasm disintegrating pieces, and the accumulation of lipid droplets intracytoplasm, the specific hepatocyte balloon degeneration performance. All these changes were mitigated by EGCG. EGCG alone did not cause morphological changes in the liver (Figure 3).

### EGCG reduced the oxidative damage induced by NaAsO_2_ in rat

**Figure 4:**
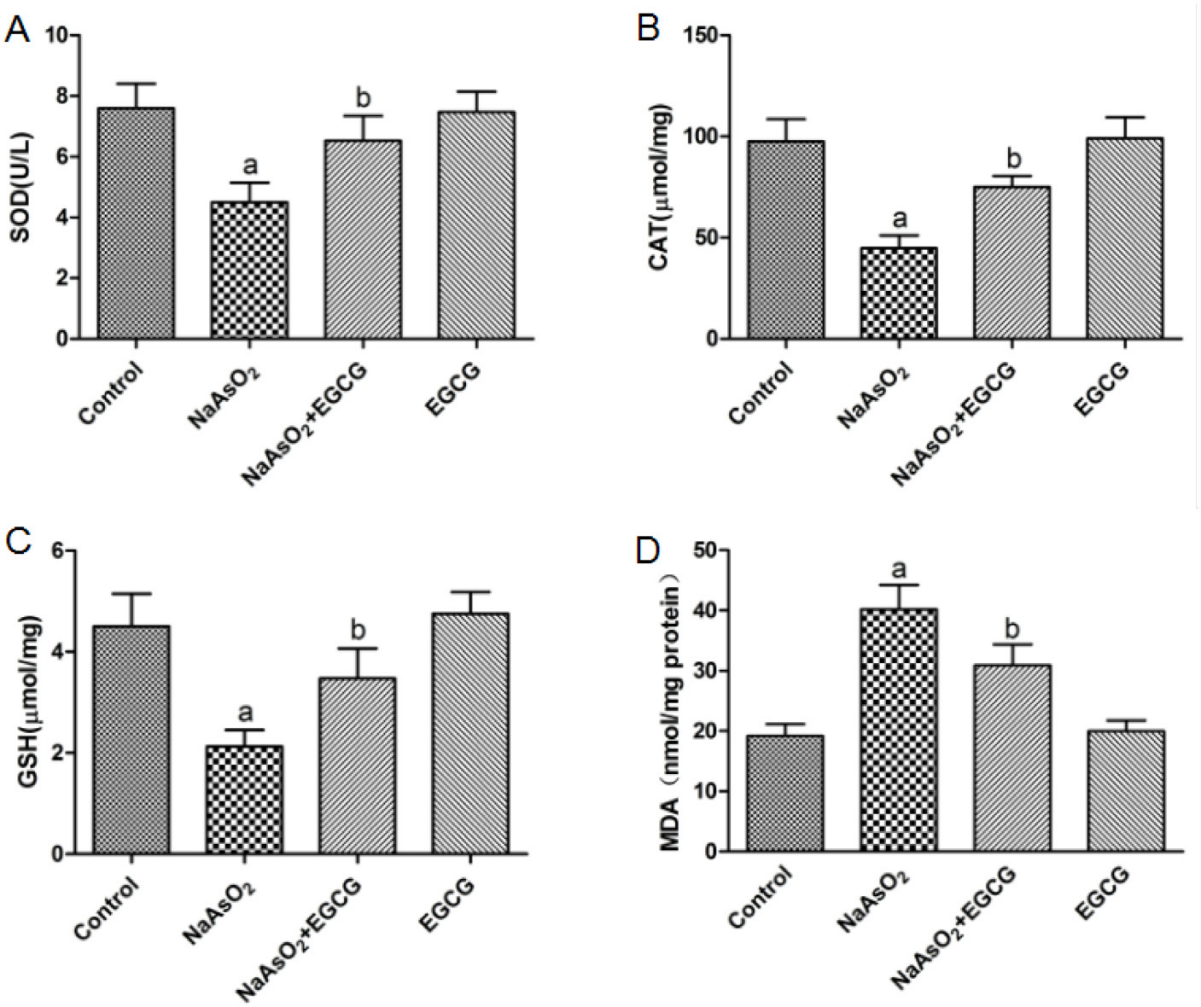
Effects of EGCG on the NaAsO_2_-induced oxidative stress of liver in rats **(A)** SOD, **(B)** CAT, **(C)** GSH, **(D)** MDA Vs control, ^a^*P*<0.05;Vs NaAsO_2_ group, ^b^*P*<0.05.

As shown in Figure 4, the activities of SOD, CAT and GSH were reduced and MDA was increased in NaAsO_2_-treated rat compared with the control group. However, treatment with EGCG caused a significant increase in SOD, CAT and GSH activity and decreased the levels of MDA compared with the NaAsO_2_-treated group. These results collectively suggested that EGCG could attenuate NaAsO_2_-induced oxidative stress (Figure 4A, 4B, 4C, 4D).

### EGCG reduced liver total arsenic concentration

**Figure 5:**
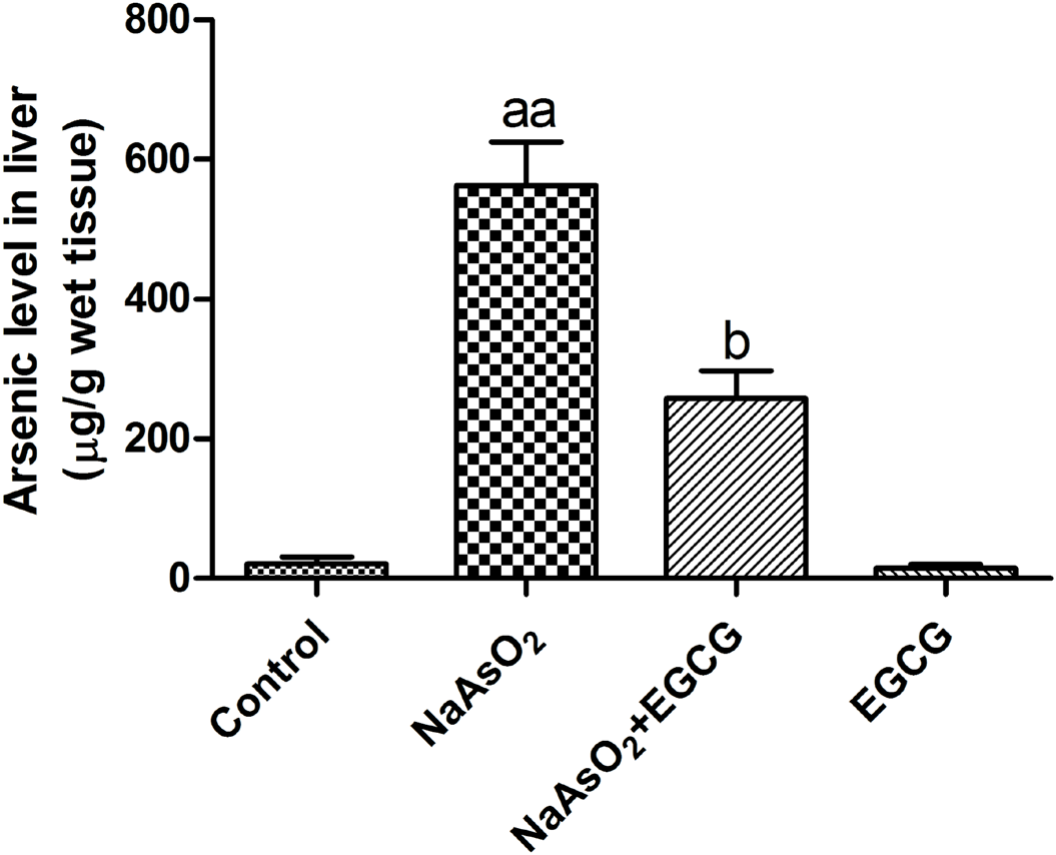
Effects of EGCG on the arsenic accumulation in the liver Vs control, ^aa^*P*<0.01; Vs NaAsO_2_ group, ^b^*P*<0.05.

To further confirm the effect of EGCG on NaAsO2 in liver, the levels of arsenic were determined. As shown in Figure 5, there existed a significant increase in the liver levels of arsenic in NaAsO_2_-treated rats. EGCG decreased obviously arsenic accumulation induced by NaAsO_2_.

### EGCG attenuated NaAsO_2_-induced injuries and decreased ROS generation in hepatocytes

**Figure 6:**
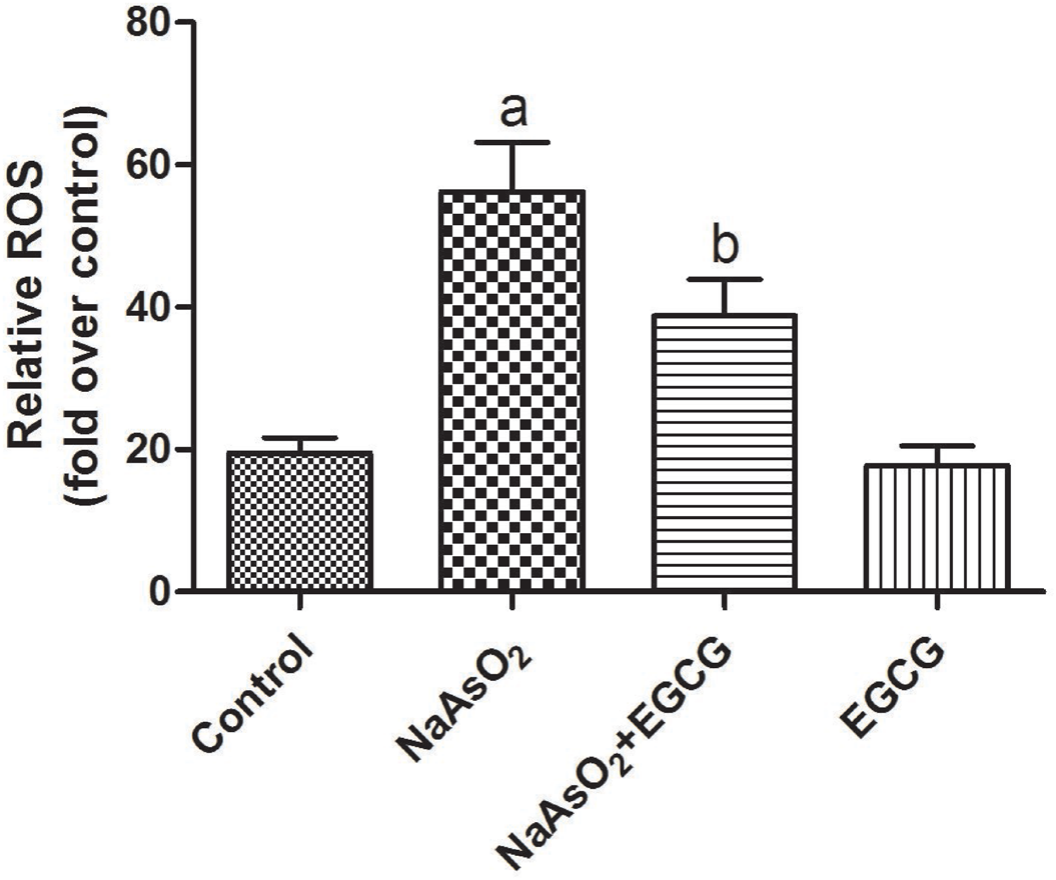
Effects of EGCG on ROS generation in rats Vs control, ^a^*P*<0.05;Vs NaAsO_2_ group, ^b^*P*<0.05.

Arsenic-related injuries have been regarded to be associated with over-production of ROS. To determine the effect of EGCG on NaAsO_2_-induced oxidative stress, we examined the intensity of total ROS by incubating hepatocytes with 10μM DCF fluorescence-AM. As shown in Figure 6, arsenic significantly increased fluorescence, indicating that NaAsO_2_ increased ROS generation in hepatocytes. Treatment with EGCG significantly decreased concentration of ROS in hepatocytes (Figure 6).

### EGCG down-regulated the expression levels of Nrf2 pathway related genes in liver tissues and hepatocytes

**Figure 7:**
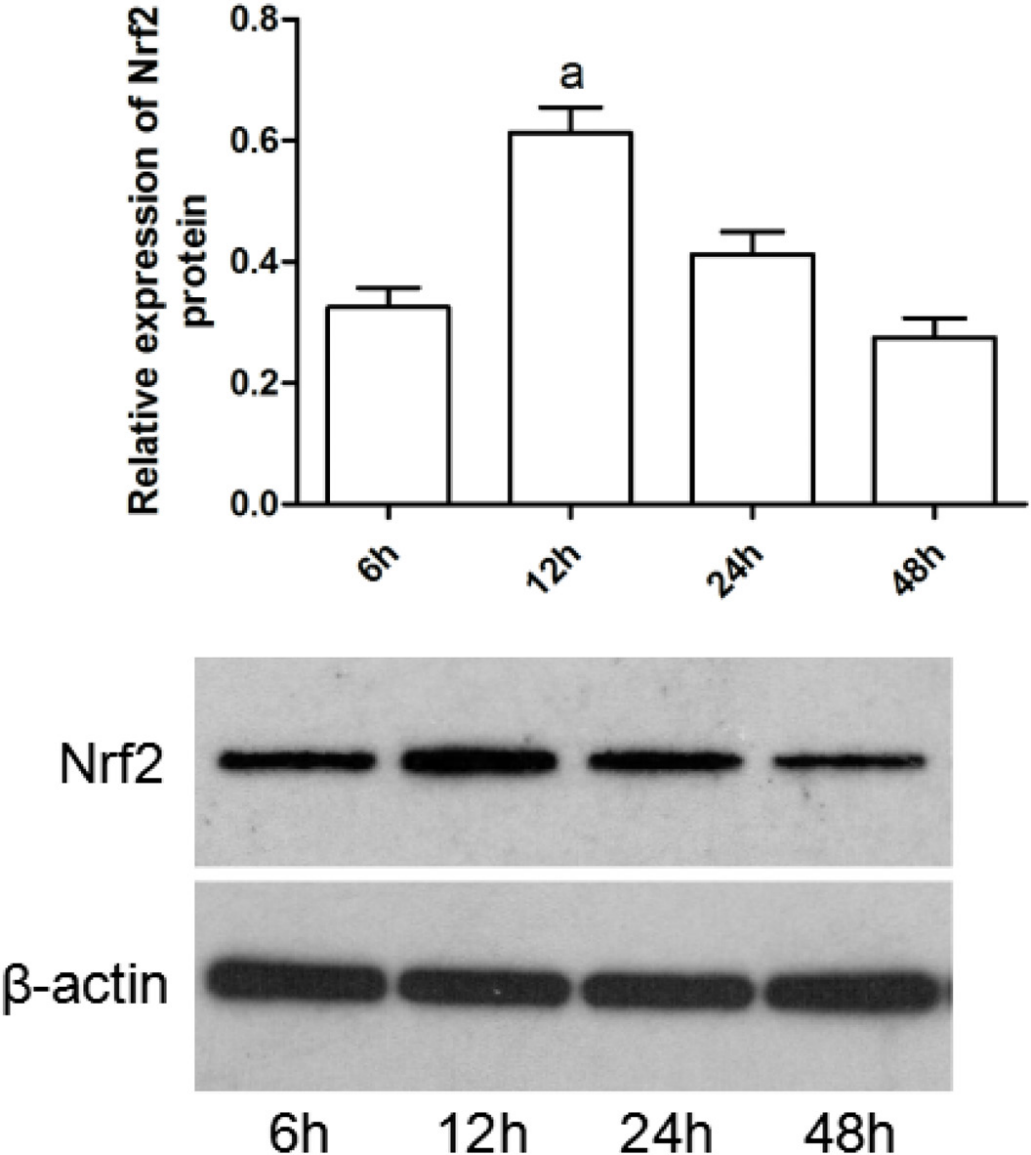
NaAsO_2_ increases total cellular Nrf2 protein levels in hepatocytes Whole-cell protein extracts were separated by SDS-PAGE, and western blot analysis was conducted using antibodies of Nrf2 and β-actin, respectively. Cells were treated with 1μmol/L of NaAsO_2_ for 6, 12, 24, and 48h. Data were mean ± SEM from three different samples. Vs control, ^a^*P*<0.05;Vs NaAsO_2_ group, ^b^*P*<0.05.

Inorganic arsenic could stimulate the activation of Nrf2 pathway. To determined the effect of EGCG on Nrf2 pathway related genes. Firstly, Nrf2 protein levels were assessed at different time interval (6h, 12h, 24h, 48h). Western blot analysis results showed that the total cellular Nrf2 protein levels obviously increased at 12 h (Figure 7), and decreased thereafter, consistently with the results of 1μmol/L NaAsO_2_ treatment in hepatocytes. Accordingly, in the subsequent experiments, we chose 12 h as the longest exposure time.

**Figure 8:**
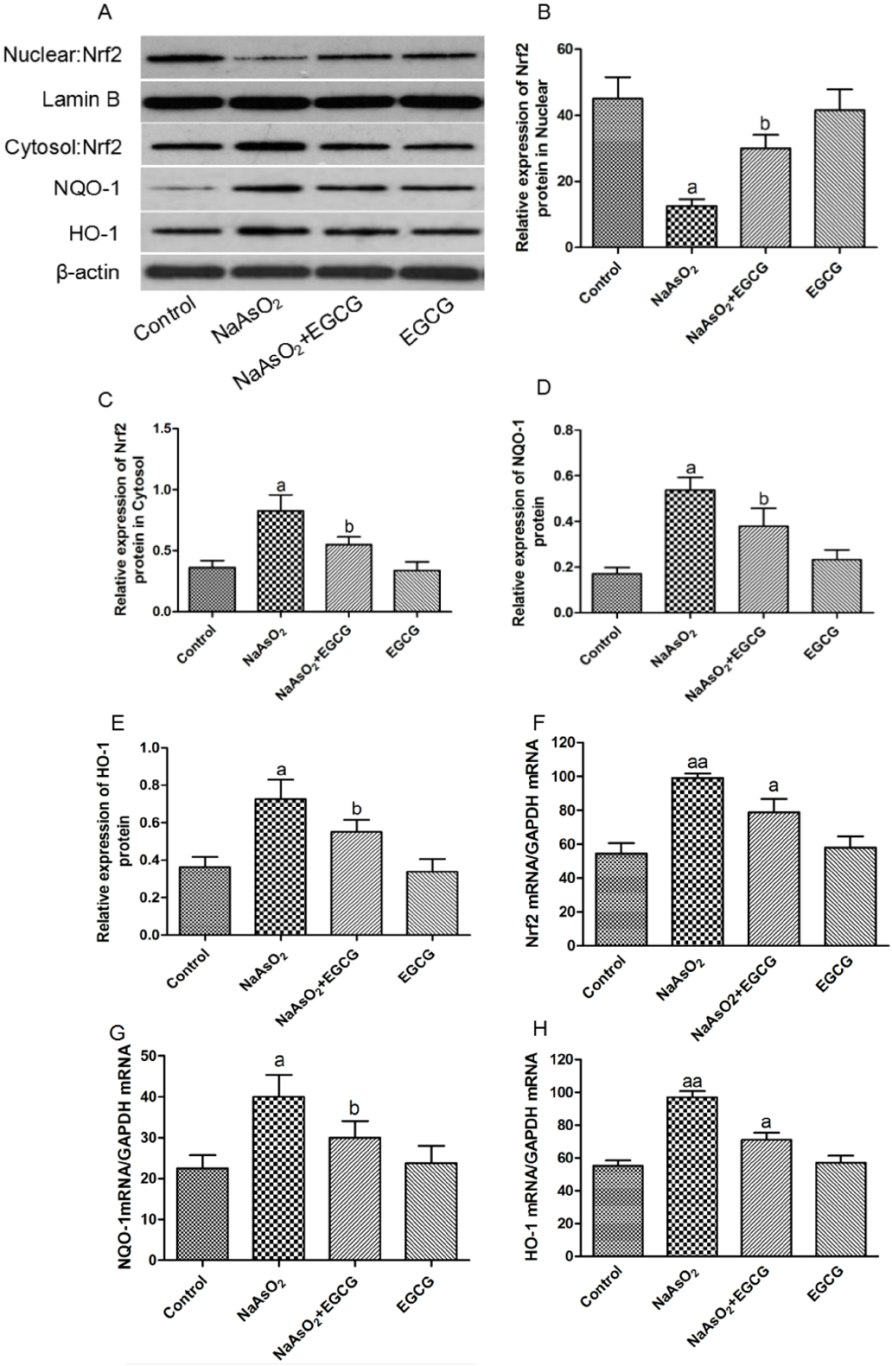
Effects of EGCG on NaAsO_2_-induced Nrf2, NQO-1 and HO-1 expressions in hepatocytes Data were mean ± SEM from three different samples. **(A)** Western blotting data showed that the translocation of Nrf2 from cytosol into nucleus using nucleus fraction and the protein expressions of Nrf2, NQO-1 and HO-1. **(B)** Relative expression of Nrf2 protein in Nucleus. **(C)** Relative expression of Nrf2 protein in Cytosol. **(D)** Relative expression of NQO-1 protein. **(E)** Relative expression of HO-1 protein. **(F)** mRNA expression of Nrf2. **(G)** mRNA expression of NQO-1. **(H)** mRNA expression of HO-1. Vs control, ^a^*P*<0.05, ^aa^*P*<0.01; Vs NaAsO_2_ group, ^b^*P*<0.05.

Activation of Nrf2 has been found to be associated with arsenic. To determine the changes of Nrf2 mRNA by NaAsO_2_ exposure. Real-time PCR and western blot results showed that NaAsO_2_ exposure caused increases in the expression of Nrf2 in hepatocytes (Figure 8). EGCG significantly inhibited the over-expression of Nrf2 mRNA and protein. EGCG alone showed no changes on expressions of Nrf2 mRNA in Chang human hepatocytes.

To further assess whether arsenic-induced Nrf2 activation could affect the expressions of Nrf2 pathway-related genes including NQO1 and HO-1, western blot analysis were applied to determine the expressions of NQO1 and HO-1 in Chang human hepatocytes. As shown in Figure 8A, 8B, 8C, 8D, 8E, arsenic moderately up-regulated NQO1 and HO-1protein and mRNA expression, EGCG down-regulated the protein expressions of NQO1 and HO-1.

We next determined the expression levels of NQO1 and HO-1 gene. As depicted in Figure 8F, 8G, 8H, arsenic obviously increased the expressions of NQO1 and HO-1 gene. Meanwhile, induction of HO-1 gene was more dramatic than NQO1, which were consistent with NQO1 and HO-1 protein expressions. EGCG inhibited the activations of these proteins in NaAsO_2_-induced hepototoxicity. These suggested that EGCG exerted protective effects on arsenic-induced hepototoxicity through inhibition of Nrf2-mediated signaling pathway.

## DISCUSSION

The herein presented study describes the effects of EGCG on the NaAsO_2-_induced Nrf2 signaling for hepatotoxicity. EGCG could attenuate the arsenic-induced hepatotoxicity through the Nrf2 signaling pathway.

Chronic arsenic exposure through polluted environment causes abnormal liver function, hepatomegaly, hepatoportal sclerosis, liver fibrosis and cirrhosis. However, accumulating evidences have revealed that the exact mechanisms by which arsenic-induced hepatotoxicity are not fully elucidated. It has been shown that oxidative stress and inflammation are the main causes in the pathogenesis of arsenic-induced organ damage [13]. Emerging evidences have support the widely accepted theory that oxidative stress is one of important mechanisms in arsenite and other arsenicals induced hepatotoxicity [14–17]. Several lines of studies have shown that arsenic could cause excessive production of ROS in various tissues [17, 18]. These oxidants enhance the generation of ROS and exhausted antioxidant defense enzymes, such as catalase, superoxide dismutase and GPx, cause oxidative stress and lead to damage in lipid. [19]. Literatures have reported that the toxic effects of arsenic are due to its ability to induce reactive oxygen species [13]. In present study, we found that NaAsO_2_ could induce oxidative stress and liver injury *in vivo* and *in vitro*. NaAsO_2_ enhanced the levels of injury markers such as ALT, AST, ALP and LDH and MDA content, showed decreases in the activities of antioxidative enzymes, including SOD, CAT, GSH compared with control group. Furthermore, noticeable morphological changes found in the liver further confirmed the adverse actions of NaAsO_2_ on the liver. These results strongly suggested that NaAsO_2_ disrupted the balance between oxidant and antioxidant agents and demonstrated NaAsO_2_-induced liver oxidative damage. Administration of EGCG apparently could attenuate the arsenic-induced hepatotoxicity including lowering the levels of transaminase, reversal the activities of SOD, CAT, GSH in liver tissue. These findings suggested that EGCG could protect liver from arsenic-induced hepatotoxicity.

Nrf2 is a basic leucine zipper redox-sensitive transcription factor that controls the redox state of cell in harmful stresses [20]. Under physiological conditions, Nrf2 is localized in the cytoplasm and anchored by its inhibitor, Keap 1. However, under conditions of oxidative stress, Nrf2 dissociates from Keap 1, translocates into the nucleus and regulates the expression of antioxidant genes including HO-1 and NQO-1 via interaction with the antioxidant response element. In addition, accumulating reports have demonstrated that Nrf2 involves in the arsenic-induced oxidative stress. Till now, Nrf2 has been proven to act as a harmful factor in many pathological processes and activated in arsenic-related hepatotoxicity [21]. These pathologies typically involve induction of genes that are regulated by Nrf2 in specific tissues. The beneficial effects of Nrf2 were identified early as a central part of the biphasic protective response induced after exposure to oxidative stress [22]. It has been demonstrated that Nrf2 activation involved in arsenic induced-cell damage including human hepatocytes, indicating that Nrf2 pathway activation by arsenic is a kind of cellular ubiquitous phenomenon, and play indispensable roles for the cellular defense against arsenic hepatotoxicity [23–25]. Recent studies have shown that expression of Nrf2 and its target genes initially increased and then gradually fell over the duration of arsenic exposure [22]. Several evidences have been depicted for arsenic exposure may activate Nrf2 pathway to maintain cell physiological function [26]. Several lines of evidence showed that Nrf2 could bind to ARE and activate its downstream target genes [27, 28]. In this study, arsenic induced Nrf2 expression, furthermore, our data showed that NQO-1, HO-1 levels up-regulate in arsenic-induced hepatotoxicity. These findings were in consistent with previous studies. However, treatment with EGCG reversed above mentioned injuries. And also these results were also consistent with up-regulation of Nrf2 target genes. Our results indicated that EGCG may serve as an enhancer of antioxidative stress.

In summary, we demonstrated that EGCG could alleviate arsenic-induced oxidative stress and liver injury through activating the Nrf2 pathway. As oxidative damage is an important toxic mechanism of arsenic and is associated with the pathological processes of diverse liver diseases, we believe EGCG could be useful in treating many liver disorders through reducing oxidative stress by activating Nrf2 pathway.

## MATERIALS AND METHODS

### Preparation of arsenic and EGCG

Arsenic dose selection in the present study was based on the previous report [13, 29]. Arsenic as sodium arsenite at a dose of 5 mg/kg bw/day was dissolved in water and treated orally. EGCG, purchased from Sigma Aldrich Corporation (Shanghai, China) with a purity of 98%, was dissolved in distilled water and each rat received daily 1 ml at a dose of 40 mg/kg bw/day administrated orally just after exposure to arsenic by intragastric intubation throughout the experimental period. The stock suspension was prepared with distilled water and stored at 4°C for two days (concentration: 50 mg/mL). Throughout all the experiments, the characteristics of all the drugs have not changed.

### Cell culture

Chang human hepatocyte line (number ZN1003, Cell Bank of Chinese Academy of Sciences, Shanghai, China) was cultured in RPMI medium 1640 (Gibco, USA) supplemented with 10% fetal bovine serum (FBS, Tianjin Bio, China) and antibiotics (100 U/mL penicillin and 100μg/mL streptomycin, Sigma, USA). All cell cultures were maintained in a humidified and 37°C incubator with 5% CO_2_ and 95% air. Cells were subcultured with 0.25% trypsin (Gibco, USA) at a ratio of 1 : 3 and used at 75%-80% confluence for the following experiments.

### Assessment of viability by MTT assay

Cell viability was determined by the MTT assay (Beyotime, Beijing, China). The cells were seeded at 1×10^4^ cells/well in 96-well plates. After treatment with control or NaAsO_2_ (1μM) or NaAsO_2_+EGCG for 12h, 20 μl of 5 mg/ml MTT solution was added to each well, and incubated for 4 h. The supernatants were aspirated, and the formazan crystals in each well were dissolved in 150 μl of dimethyl sulfoxide. The absorbance was measured at 570 nm using a micro plate reader (Spectrafluor, TECAN, sunrise, Austria).

### Ethics statement

All animal experiments were performed according to institutional ethical committee guidelines and approved by the Medical Ethics Committee of Central South University and were in accordance with the US National Institutes of Health Regulations for the Care and Use of Animals (NIH Publication No.85-23, revised 1996). All efforts were made to minimize sufering.

### Animals and pharmacological treatment *in vitro* and experimental protocol

Adult male albino rats of Male Sprague-Dawley (SD) weighing 180g to 220 g were used for the experiment. The animals were housed in polypropylene cages and maintained in 12-h light/12-h dark cycle, 50% humidity and 25 ± 2°C. The animals had free access to standard pellet diet (M/S. Pranav Agro Industries Ltd., Bangalore, India) and water adlibitum. This study was approved by Institutional Animal Ethics Committee of Central South University and the study was conducted in accordance with the “Guide for the Care and Use of Laboratory Animals”.

All animals were randomly assigned to the following groups with 10 animals/group as followed: control (saline), NaAsO_2_ (5 mg/kg/day), NaAsO_2_+EGCG (dissolved in saline, 50 mg/kg/day, i.g.) and EGCG (dissolved in saline, 50 mg/kg/day, i.g.). The rats of control group and EGCG group were supplied with saline for 30 days. Rats in the NaAsO_2_ group and NaAsO_2_+EGCG group were supplied with NaAsO_2_-containing drinking water (50 mg/kg) for 30 days. On 31 day, the rats were euthanized for following experiments. At the end of the experimental period, the animals in different groups were given by an intraperitoneal injection of Chloral hydrate (0.03 mg/kg), then opened the chest of animal. Blood samples were collected through heart puncture in two different tubes. One is heparinised, for plasma and another without heparin for serum collection. Serum and plasma were separated by centrifugation and used for various biochemical estimations.

### Determination of tissue hepatotoxicity enzymatic indices

Tissue samples were assayed for the determination of liver enzymes (AST: Aspartate transaminase, ALT: alanine aminotransferase, alkaline phosphatase (ALP) and oxidative stress relatedenzymes (CAT: Catalase, MDA: malonyldialdehyde, SOD: superoxide dismutase and GSH: Glutathione) by kits using an automatic chemical analyzer (7060, Hitachi, Tokyo, Japan) according to manufacturer’s instructions. The tissues were removed, rinsed with physiological saline and immediately weighed. All the samples were stored at -70°C throughout the experimental period.

### Measurement of ROS production

Measurement of mitochondrial ROS was performed as previously described. Hepatocyte from each group were suspended in PBS and incubated with 50μM 2’,7’-dichlorofluoresin diacetate (DCFH-DA) in modified Krebs solution (135 mM NaCl, 5.9 mM KCl, 1.5 mM CaCl_2_, 1.2 mM MgCl_2_, 11.5 mM glucose, 11.6 mM HEPES, pH 7.4) supplemented with 0.1% bovine serum albumin and 0.02% Pluronic F127 at 37 °C for 1 hour in the dark at room temperature. ROS generation was recorded at excitation wavelength 488 nm and emission wavelength 525 nm using fluorescence microscopy.

### Determination of arsenic accumulation in the liver

The arsenic contents in hepatic tissues of all rats were analyzed following the method as previously described with an atomic fluorescence spectrometry system.

### Histopathological studies

The liver was isolated immediately after sacrificing the animal and washed with ice-cold normal saline, trimmed and embedded in parain and sliced at 4μM thickness. Then the sections were stained with hematoxylin and eosin (HE). The structure was then examined under a light microscope by a pathologist blinded to the groups under study.

### Nuclear extract preparation

Nuclear extracts for western blotting was prepared by Nuclear/Cytosol Fractionation Kit (Biovision, Milpitas, CA, USA). Cells were washed with ice-cold PBS and collected by centrifugation at 600×g for 5 min at 4°C. We resuspended cell pellets in 0.2 mL cytosol extraction buffer A containing DTT and protease inhibitors and kept on ice for 10 minutes. The supernatant obtained was added to 11 μL of cytosol extraction buffer B, maintained on ice for 1 min, and centrifuged at 16,000×g for 5 min. The supernatant (cytosol extract) was transferred to a new tube. The pellet (contains nuclei) was resuspended in 100 μL of nuclear extraction buffer mix, vortexed on ice for 15 seconds, and then centrifuged at 16,000×g for 10 min. The supernatant (nuclear extract protein) concentration was measured using the Bradford method for western blotting.

### Preparation of protein extracts and western blot assay

Protein was extracted from hepatocyte and liver tissue with RIPA buffer (containing 0.1% PMSF), and equal amounts of protein from each sample (50μg) were separated by 10% or 12% SDS/PAGE and transferred to polyvinylidene fluoride membranes. The membranes were then incubated with primary antibodies overnight at 4°C, and horseradish peroxidase (HRP)-coupled goat anti-mouse or goat anti-rabbit secondary antibody (sc-2005, 1:2000, sc-2030, 1:5000; Santa Cruz, CA, USA). The chemiluminescence signals were detected with the EasySee Western Blot Kit (Beijing TransGen Biotech, Beijing, China). The densitometric analysis was conducted with Image J 1.43 (National Institutes of Health). Primary antibodies used and their dilution are shown as follows: Nrf2 (1 : 1000), NQO1 (1 : 1000), and HO-1 (1 : 1000), anti-β-actin (1:1000). Blots were then incubated with chemiluminescence reagents (PicoWest Super Signal, Pierce Biotechnology, IL, USA) and visualized using Electrophoresis Gel Imaging Analysis System (MF-ChemiBIS 3.2, DNRBio-Imaging Systems, Israel).

### Total RNA isolation and quantifcation of mRNA by RT-PCR

Total RNA was isolated using RNAzol B reagent (Tel-Test, Inc., Friendswood, TX, USA) according to the manufacturer’s protocol. The concentration of total RNA in each sample was quantified spectrophotometrically at 260 nm. The integrity of each RNA sample was evaluated by for maldehyde-agarose gel electrophoresis before analysis.

Total RNA was reverse-transcribed into cDNA by High Capacity cDNA Archive Kit (Applied Biosystems, FosterCity, CA, USA), and the resulting cDNA was used for real-time PCR analysis using Power SYBR Green PCRMaster Mix in a 7900HT Fast Real-Time PCR System (Applied Biosystems, Foster City, CA, USA). Oligonucleotide primers were designed with Primer3 software.

### Statistical analysis

Data were expressed as mean ± SEM and analyzed using a one-way ANOVA followed by Duncan’s multiple range test utilizing SPSS 18.0 Software (SAS, NC). The significant level was set at *P*<0.05.

## Abbreviations

EGCG: (-)-Epigallocatechin-3-gallate
Nrf2: nuclear factor erythroid 2-related factor 2
HO-1: heme oxygenase-1
CAT: Catalase
MDA: malonyldialdehyde
SOD: superoxide dismutase
GSH: Glutathione
MTT: 3-(4,5-dimethylthiazol-2- yl)-2,5- diphenyltetrazolium
